# How do I diagnose Maturity Onset Diabetes of the Young in my patients?

**DOI:** 10.1111/cen.14744

**Published:** 2022-05-02

**Authors:** Kevin Colclough, Kashyap Patel

**Affiliations:** ^1^ Exeter Genomics Laboratory Royal Devon & Exeter NHS Foundation Trust Exeter UK; ^2^ Institute of Biomedical and Clinical Science University of Exeter Medical School Exeter UK

**Keywords:** biomarkers, delivery of health care, genetic testing, Maturity Onset Diabetes of the Young, next‐generation sequencing, patient selection, precision medicine

## Abstract

Maturity Onset Diabetes of the Young (MODY) is a monogenic form of diabetes diagnosed in young individuals that lack the typical features of type 1 and type 2 diabetes. The genetic subtype of MODY determines the most effective treatment and this is the driver for MODY genetic testing in diabetes populations. Despite the obvious clinical and health economic benefits, MODY is significantly underdiagnosed with the majority of patients being inappropriately managed as having type 1 or type 2 diabetes. Low detection rates result from the difficulty in identifying patients with a likely diagnosis of MODY from the high background population of young onset type 1 and type 2 diabetes, compounded by the lack of MODY awareness and education in diabetes care physicians. MODY diagnosis can be improved through (1) access to education and training, (2) the use of sensitive and specific selection criteria based on accurate prediction models and biomarkers to identify patients for testing, (3) the development and mainstream implementation of simple criteria‐based selection pathways applicable across a range of healthcare settings and ethnicities to select the most appropriate patients for genetic testing and (4) the correct use of next generation sequencing technology to provide accurate and comprehensive testing of all known MODY and monogenic diabetes genes. The creation and public sharing of educational materials, clinical and scientific best practice guidelines and genetic variants will help identify the missing patients so they can benefit from the more effective clinical care that a genetic diagnosis brings.

## 1 WHAT IS MODY?

1.1

The term Maturity Onset Diabetes of the Young (MODY) describes a form of monogenic diabetes (i.e., diabetes caused by a defect in a single gene) that is characterized by young onset, non‐insulin‐dependent diabetes in slim individuals transmitted in an autosomal dominant manner.^1^ MODY differs from other types of monogenic diabetes in that it is diagnosed outside of the neonatal period and there is no extra‐pancreatic developmental disease characteristic of a syndrome.

MODY is typically diagnosed before the age of 30 years. In contrast to type 1 diabetes, MODY patients rarely have a severe presentation with ketoacidosis and weight loss, do not have islet autoantibodies, have significant endogenous insulin production outside of the honeymoon period and are very likely to have a parent affected with diabetes.^2^ They differ from the majority of type 2 diabetes patients by their young age of diagnosis, nonobesity and absence of insulin resistance/dyslipidaemia.^3^

MODY is both clinically and genetically heterogeneous, with 10 different genetic subtypes identified to date (Table 1).^31^ Genetic testing of all MODY genes is essential to determine the genetic subtype as this in turn determines the patient's clinical features, clinical course and response to treatment. The majority of patients with a genetic a diagnosis of MODY (>80%) will have mutations in the *HNF1A*, *HNF4A* or *GCK* genes,^32^ and a genetic diagnosis of these subtypes has important implications for precision medicine‐based clinical care.^33^ This article will therefore focus on these three subtypes.

**Table 1:** The different genetic subtypes of MODY.

Gene	Clinical characteristics	Proportion of patients with suspected MODY and a genetic diagnosis^a^	Mode of inheritance	Mutational mechanism	References
Genes associated with the classical MODY phenotype					
*HNF1A*	Progressive insulin secretion defect with patients presenting as teenagers or young adults. Sensitive to low‐dose sulphonylurea treatment. Glycosuria due to a low renal threshold for glucose. Neonatal hypoglycaemia reported in some patients. Increased risk of cardiovascular mortality despite a protective lipid profile.	33%	Autosomal dominant, very rarely recessive if mutations are hypomorphic for example p.Ala251Thr.	Loss of function (haploinsufficiency) resulting in an insulin secretion defect.	[4, 5, 6]
*HNF4A*	Progressive insulin secretion defect with patients presenting as teenagers or young adults. Sensitive to low‐dose sulphonylurea treatment. Hyperinsulinism occurs *in utero* resulting in increased birthweight and risk of neonatal hypoglycaemia. The p.Arg63Trp mutation also causes a proximal tubulopathy and the p.Arg114Trp mutation is associated with a significantly reduced penetrance for diabetes.	14%	Autosomal dominant.	Loss of function (haploinsufficiency) resulting in an insulin secretion defect.	[4, 7, 8]
*GCK*	Glucose sensing is reset to a higher level resulting in mild fasting hyperglycaemia from birth (typically in the range of 5.5–8 mmol/L with HbA1c 40–60 mmol/mol) and small postprandial increase in glucose (<3 mmol) that does not increase risk of diabetes complications. Usually asymptomatic so often detected incidentally for example in pregnancy. Hyperglycaemia does not respond to or require treatment outside of pregnancy; treatment during pregnancy is determined by the *GCK*mutation status of the foetus.	22%	Autosomal dominant, although rare recessive cases occur with specific mutations that can result in a more severe phenotype similar to HNF MODY.	Loss of function (haploinsufficiency) causing a defect in glucose sensing by the beta cell.	[9, 10]
*INS*	Highly penetrant subtype that resembles type 1 diabetes without autoimmunity. Insulin treatment can help reduce ER stress on beta cell and preserve insulin secretion.	2%	Autosomal dominant.	Toxic gain of function from misfolded proteins causing ER stress and beta cell death. The p.Arg46Gln mutation specifically causes diabetes due to loss of insulin activity.	[11, 12]
*ABCC8*	Diabetes may occur as the relapsing stage of TNDM, or as isolated MODY when NDM is not penetrant. Patients with activating mutations are sensitive to sulphonylurea treatment, but not those with inactivating mutations.	4%	Autosomal dominant.	Gain of function (activating) missense mutations. Rarely, specific dominant loss of function (inactivating) missense mutations associated with congenital hyperinsulinism may cause MODY with reduced penetrance but the mechanisms driving this are not understood.	[13, 14]
*KCNJ11*	Diabetes may occur as the relapsing stage of TNDM, or as isolated MODY when NDM is not penetrant. Patients are sensitive to sulphonylurea treatment.	2%	Autosomal dominant.	Gain of function (activating) missense mutations.	[15]
*RFX6*	Significantly reduced penetrance for diabetes. Hypothetical response to DPP4 inhibitors or GLP1 receptor agonists based on observation of lower GIP levels.	3%	Autosomal dominant.	Loss of function (haploinsufficiency). Nonsense, frameshift and splicing variants resulting in a null allele. Missense variants are yet to be associated with MODY.	[16]
*PDX1*	Diabetes with reduced/variable penetrance that is rarely treated with insulin.	<1%	Autosomal dominant.	Combination of haploinsufficiency and dominant negative effects. Specific frameshift mutations trigger translation reinitiation, generating two mutant proteins that lack either the DNA binding domain or the transactivation domain.	[17, 18]
*NEUROD1*	Diabetes with reduced/variable penetrance that is rarely treated with insulin.	1%	Autosomal dominant.	Unclear—possible haploinsufficiency although specific missense mutations in the DNA binding domain may act in a dominant negative manner.	[19, 20]
Genes associated with syndromic subtypes of monogenic diabetes that may result in referral for MODY genetic testing in the absence of typical characteristic nondiabetic features					
*HNF1B*	Renal structural disease, urogenital tract malformations, hypomagnesaemia, gout, abnormal liver function, pancreatic hypoplasia, autism (*HNF1B*deletion cases). Severity of the renal phenotype is highly variable and patients may present with isolated MODY.	6%	Autosomal dominant.	Loss of function (haploinsufficiency). Whole gene deletions of *HNF1B*account for ~50% of all cases.	[21]
*MT‐TL1*	Bilateral sensorineural deafness. Penetrance of deafness is highly variable due to variation in heteroplasmy in specific tissues and patients may present with isolated MODY.	8%	Mitochondrial (maternal).	m.3243A>G mutation.	[22]
*CEL*	Pancreatic lipomatosis and exocrine dysfunction.	<1%	Autosomal dominant.	Toxic gain of function from misfolded proteins caused by frameshift variants in the first 1–4 repeats of the VNTR region.	[23]
*PAX6*	Aniridia.	<1%	Autosomal dominant.	Loss of function (haploinsufficiency).	[24]
*WFS1*	Optic atrophy, deafness, bladder dysfunction, neurological problems. Islet autoantibody negative diabetes is usually the first presenting feature in childhood and this may trigger referral for MODY testing in the absence of any other features.	2%	Autosomal recessive. A specific missense mutation, p.Trp314Arg, causes autosomal dominant nonsyndromic *WFS1*‐related diabetes.	Loss of function.	[25, 26]
*GATA6*	Structural heart defects, pancreatic agenesis and neonatal diabetes. Very rarely patients can present with diabetes in childhood without structural defects of the heart and pancreas.	<1%	Autosomal dominant.	Loss of function (haploinsufficiency). Typically null mutations are identified and can arise *de novo*.	[27]
Putative MODY genes with limited evidence for gene‐disease association					
*APPL1*	Associated with a later age of onset, less severe disease and reduced penetrance for diabetes. Potentially risk variants for type 2 diabetes in combination with obesity rather than highly penetrant monogenic mutations.	<1%	Autosomal dominant.	Loss of function (haploinsufficiency).	[28]
Genes with refuted evidence for gene‐disease association					
*PAX4*	Potentially risk variants for type 2 diabetes in combination with obesity rather than highly penetrant monogenic mutations.	<1%	Autosomal dominant.	Loss of function (haploinsufficiency).	[29]
*BLK*					
*KLF11*					

*Note*: Genes are categorized into those that cause the classic MODY phenotype of isolated diabetes, those where the diabetes can resemble MODY but is part of a syndrome with characteristic nondiabetic features, and putative genes published as likely to cause MODY but require more evidence to definitively assign MODY gene status. *HNF4A* mutations are described using the reference sequence NM_175914.4 and may differ from descriptions in the literature.

Abbreviations: ER, endoplasmic reticulum; MODY, Maturity Onset Diabetes of the Young;  NDM, neonatal diabetes mellitus; TNDM, transient neonatal diabetes mellitus; VNTR, variable number tandem repeat.

^a^ Exeter of 297 patients with a genetic diagnosis of monogenic diabetes tested using targeted next generation sequencing of all known MODY genes due to a suspected diagnosis of MODY.

*HNF1A* and *HNF4A* encode transcription factors that regulate the expression of genes involved in insulin secretion in the beta cell. Loss of HNF1A or HNF4A results in progressive young‐onset diabetes requiring treatment to prevent complications. These patients are highly sensitive to the glucose lowering effects of sulphonylureas and may experience hypoglycaemia even at low doses.^34^ Foetuses that inherit an *HNF4A* mutation have an increased risk of macrosomia and hyperinsulinaemic hypoglycaemia at birth.^35^ In contrast, *GCK* mutations result in a glucose sensing defect in the beta cell that results in mild, persistent fasting hyperglycaemia from birth. Since the defect is in glucose sensing rather than regulation, the beta call counter‐regulates any attempts to reduce glucose levels and a stable raised glucose and HbA1c level is maintained. The GCK phenotype is strikingly similar between patients; fasting hyperglycaemia in the range of 5.5–8 mmol/L, HbA1c 40–60 mmol/L and a small postprandial increase in glucose (typically <4 mmol/L difference between fasting and 2 h values on OGTT) because the expression of the unaffected gene copy is upregulated to compensate for the loss the activity of the defective gene.^36^ Because the hyperglycaemia is asymptomatic, the majority of patients are identified only when fasting blood glucose is measured, most often during pregnancy.

Patients referred for MODY testing may also have mutations in genes that cause syndromic diabetes, but the diabetes phenotype is similar to MODY and they either lack the clinical features of the syndrome because of variable expressivity, severity and penetrance, or the clinician is unaware of the significance of the features and they are not reported to the testing laboratory.^37^ Mutations in the *HNF1B* gene (associated with a syndrome of diabetes and structural kidney disease), or the mitochondrial *MT‐TL1* gene mutation m.3243A>G (associated with a syndrome of diabetes and sensorineural deafness) account for ~15% of the patients referred for MODY testing that receive a genetic diagnosis.^38^ Patients can also be unexpectedly diagnosed with rarer conditions such as Wolfram syndrome where diabetes can be the presenting feature and the patient is tested before the onset of syndromic features.^37^

Estimates of MODY prevalence will vary depending on the clinical selection criteria, population tested and the test methodology used. The most accurate estimates are those in paediatric/young adult populations where comprehensive testing of all known MODY genes has been undertaken in patients with negative islet autoantibodies and detectable C‐peptide. It is thought that MODY accounts for about 2%–4% of all diabetes in children and young adults.^40^, ^41^

## 2 WHY SHOULD I BE REQUESTING MODY GENETIC TESTING FOR MY PATIENTS?

Monogenic diabetes is a good example of precision medicine—the tailoring of clinical care to the individual characteristics of a specific subgroup of patients. It is the different treatment responses and clinical course in different MODY genetic subtypes that drives the need to genetically characterize these patients. Each subtype is defined by the gene containing the causative mutation, and within genes there exists specific variantphenotype relationships (see Table 1). Diagnosing a specific subtype not only informs the management of hyperglycaemia, but also provides information on clinical course and the occurrence of other associated clinical features. And health economics studies show that systematic screening for MODY is cost‐effective through reductions in treatment costs, monitoring and complication rates.^41^ The best‐known examples of precision medicine in MODY are seen in patients with HNF1A, HNF4A and GCK MODY.

Patients with HNF1A and HNF4A MODY are highly sensitive to sulphonylurea agents. A randomized trial showed a fourfold decrease in blood glucose in HNF1A MODY patients compared to matched type 2 diabetes controls.^34^ Longer term follow‐up studies show patients can successfully transfer from insulin to sulphonylurea and maintain good glycaemic control for many years, and that the factors predicting successful transfer are a shorter duration of diabetes and a lower body mass index (BMI).^42^ Therefore, early genetic diagnosis, treatment with sulphonylurea and a healthy lifestyle are essential for preventing complications. Qualitative studies have shown the dramatic positive impact that a diagnosis can have on quality of life; patients that have discontinued insulin treatment after many years describe feeling like they no longer have diabetes.^43^

Transfer from insulin to a sulphonylurea should only be attempted once a genetic diagnosis has been confirmed and C‐peptide testing has been undertaken to provide evidence of endogenous insulin production. The patient should be advised this is a ‘trial’ off insulin and if unsuccessful, insulin will need to be recommenced (particularly in older patients with long duration of diabetes). HbA1c should be measured, then insulin should be stopped and a low dose started (e.g., Gliclazide 40 mg once daily) and increased as necessary. The patient should be made aware of the signs of hypoglycaemia since this can be triggered even with very low doses.

Patients can use sulphonylurea alongside insulin at lower doses or with dipeptidyl peptidase‐4 (DPP‐4) inhibitors and glucagon‐like peptide‐1 receptor agonists (GLP‐1 RA) if sulphonylurea alone is not tolerated. Smaller randomized, double‐blind, crossover trials with GLP‐1 RA have shown reductions in plasma glucose in HNF1A MODY patients, either when used alone or in combination with DPP‐4 inhibitors.^44^, ^45^ Although the glucose lowering effect of GLP‐1 RA is smaller compared to sulphonylurea, the frequency of hypoglycaemic episodes is significantly lower. Improved glycaemic control with fewer hypoglycaemic episodes has also been reported in HNF4A MODY patients with long duration diabetes after switching from sulphonylurea to GLP‐1 RA.^46^ Another potential benefit to using GLP‐1 RA is the ability of the drug to decrease the risk of cardiovascular disease.^47^ HNF1A MODY patients have a similar risk of all‐cause mortality and cardiovascular disease to those with type 2 diabetes^48^ and may benefit from GLP‐1 RA used in combination with sulphonylurea and statin therapy.

SGLT‐2 inhibitors have also been shown to reduce blood glucose in HNF1A MODY but cause higher glycosuria compared to patients with type 2 diabetes.^49^ The impaired HNF1A‐mediated expression of SGLT2 in HNF1A MODY is likely to contribute to this^50^ and studies are required to assess the long‐term efficacy and safety with regard to risk of ketogenesis. Details of ongoing clinical trials for MODY can be found at https://clinicaltrials.gov/.

In addition to hyperglycaemia in childhood/young adulthood, foetuses that inherit an *HNF4A* mutation from either parent develop hyperinsulinism *in utero*. This results in an increase in birth weight by an average of 800 g and 10% of babies will have transient hyperinsulinaemic hypoglycaemia at birth.^35^ Knowledge of an *HNF4A* mutation in either parent therefore has implications for the management of the pregnancy; 50% of foetuses will inherit the mutation and be at risk of macrosomia and hyperinsulinism. Monitoring of foetal growth and checking for hypoglycaemia at birth is essential to prevent complications in HNF4A pregnancies.

In contrast to the severe and progressive beta cell defect seen in HNF1A and HNF4A MODY, patients with GCK‐related fasting hyperglycaemia retain their counter‐regulatory response to an increase in blood glucose concentration. Therefore they do not require treatment, and do not respond to oral or low dose insulin therapies; any attempt to lower blood glucose is counter‐regulated by the beta cell.^51^ The lifelong stable hyperglycaemia in these patients does not increase the risk of severe microvascular and macrovascular disease.^52^ Therefore, it is safe to discontinue all glucose lowering therapies, blood glucose monitoring and screening for complications. In essence these patients do not have diabetes and the GCK MODY diagnosis prevents unnecessary clinical intervention. The exception is when GCK MODY coexists with polygenic type 1 or type 2 diabetes; treatment will be required to normalize glucose and reduce complication risk, but if counter‐regulatory mechanisms are still intact then glycaemic targets should take into account the raised set point for glucose homoeostasis.

The management of GCK MODY in pregnancy is more complex and is dependent on foetal genotype status.^53^ If the foetus does not inherit the mutation, the foetal pancreas will sense the maternal hyperglycaemia and increase insulin secretion, leading to increased foetal growth and birth weight. If the foetus inherits the mutation, it will sense the maternal hyperglycaemia as normal (since the foetal pancreas will have the same glucose sensing defect) and there will be no increased foetal insulin secretion. Therefore, pregnancies where the foetus has inherited the mutation can be discharged from high‐risk antenatal care with no treatment of maternal hyperglycaemia required. An early delivery at 38 weeks or treatment of maternal hyperglycaemia with insulin can be considered when a foetus has not inherited the mutation. The challenge is that GCK MODY is an inappropriate disorder for performing high risk invasive prenatal testing, and foetal ultrasound growth scans are a highly inaccurate proxy for knowing the foetal genotype. Fortunately, noninvasive prenatal testing for GCK mutations is possible by isolating and testing cell free foetal DNA (cffDNA) from a maternal peripheral blood sample. This requires a highly sensitive and accurate bespoke assay for each mutation (e.g., droplet digital polymerase chain reaction [PCR]) but is now possible as a routine diagnostic test.^54^

The use of continuous glucose monitoring (CGM) to manage glucose control is rapidly growing and can be attributed to improved sensor accuracy, greater convenience and ease of use, and increasing availability to patients. Studies using CGM in patients with MODY are small in number and size, but they show the potential for this technology to improve clinical care. CGM can be used to assess the response to specific treatments such as sulphonylurea therapy,^55^ identify patterns of prolonged hypoglycaemia in HNF4A MODY^56^, ^57^ and can improve the efficacy of insulin therapy in GCK MODY pregnancy.^58^ CGM may also be a useful tool for studying glycaemic patterns in MODY patients with atypically severe hyperglycaemia suggestive of additional polygenic diabetes.^59^

A MODY diagnosis is important not just for the index case; it unlocks the possibility of genetic testing for the familial mutation in their family members. Affected relatives can be tested to confirm a monogenic aetiology for their diabetes, and predictive testing can be offered to asymptomatic relatives after appropriate genetic counselling to determine their future risk of developing diabetes.^60^

## 3 IF DIAGNOSING MODY IS IMPORTANT, WHO SHOULD I REFER FOR MODY GENETIC TESTING?

This is the most important question for clinicians diagnosing MODY in paediatric and young adult populations—how to identify patients for MODY genetic testing from the much higher background prevalence of young onset polygenic type 1 and type 2 diabetes. There are no simple clinical criteria that will accurately identify all MODY patients, and there is overlap with young onset polygenic type 1 and type 2 diabetes with regard to age of diabetes onset, BMI, history of parental diabetes, HbA1c levels and treatment. The traditional clinical diagnostic criteria for MODY (diagnosed <25 years, not insulin treated and a parent affected with diabetes) results in a genetic diagnosis in less than half of cases.^32^ Diagnosis is further hindered by the fact that within routine diabetes care there is a lack of genetics training and awareness of MODY, with an emphasis on treatment rather than diagnosis. As a result, the majority of MODY patients go unrecognized and are managed suboptimally.^61^

Diabetes in children is predominantly autoimmune mediated, and the most common consideration is the discrimination between type 1 diabetes and MODY. Islet autoantibodies are a highly sensitive and specific biomarker of type 1 diabetes; they are detected in ~90% of children with type 1 diabetes but are detected in only 1% with a genetic diagnosis of MODY.^62^ Systematic screening strategies for MODY in paediatric populations using antibody tests are therefore highly effective; in the ~10% of children with negative antibodies at diagnosis, approximately one in seven will have a genetic diagnosis of MODY.^2^ Positivity for a single antibody should be exclusion criteria for MODY testing. A minimum of three antibodies should be tested—GAD, IA2 and ZnT8 are preferred. IAA is not widely performed and cannot be used once insulin treatment is given, and ICA antibody testing using primate or rodent pancreatic tissue should not be performed due to a high false‐negative rate. Clinicians should seek additional antibody testing if only GAD and/or ICA are offered. Laboratories should determine the thresholds for antibody positivity based on centiles derived from nondiabetic and type 1 diabetes populations to avoid false‐positive results.

C‐peptide testing to identify and exclude those with absolute insulin deficiency (i.e., urine C‐peptide <0.2 nmol/mmol or serum/plasma <200 pmol/L) will further improve selection, but the test has limited use at diagnosis due to the preserved insulin secretion during the honeymoon period and is most useful 3–5 years postdiagnosis.^63^ The practicalities of urine C‐peptide are straightforward—a random nonfasting urine sample taken into a boric acid tube will be stable for 3 days, can be taken at home and is comparable to gold standard serum testing.^64^

Where sufficient resources are available, testing all children with negative antibodies and detectable C‐peptide will be a highly sensitive systematic approach to diagnosing MODY in paediatric clinics, identifying 99% of cases. In patients diagnosed under the age of 30 years, testing patients with negative antibodies and detectable C‐peptide identifies MODY in one in five patients tested.^40^ If necessary, specificity can be improved by testing only those patients with a parent affected with diabetes, and/or those with less severe hyperglycaemia at diagnosis. MODY patients have a milder presentation with lower HbA1c and lower incidence of ketoacidosis and osmotic symptoms. Having negative antibodies, detectable C‐peptide, a parent affected and/or an HbA1c <7.5% (58 mmol/L) at diagnosis identifies MODY in one in three children tested and would detect about 94% of cases.^2^ Therefore, where resources are available, the systematic testing of all patients diagnosed under 30 years with negative antibodies and detectable C‐peptide is an excellent strategy. Our increasing understanding of the polygenic contribution to type 1 diabetes susceptibility has led to the development of a polygenic risk score that can also discriminate MODY from type 1 diabetes and is useful in cases where the type 1 diabetes phenotype is atypical.^65^

For patients that are not insulin treated or have detectable C‐peptide 5 years postdiagnosis, continuous variables such as age of diagnosis and BMI are more informative. Selection is more accurate when using a probability model rather than absolute cut‐offs. The MODY probability calculator devised by the University of Exeter is a CE marked device that predicts the likelihood of a genetic diagnosis with high sensitivity and specificity, and outperforms human experts.^66^ The calculator takes information on sex, age at diabetes diagnosis, age at time of genetic testing, BMI, initial and current treatment, time to insulin treatment, HbA1c and parental diabetes status and uses a linear regression model to discriminate MODY from type 1 or type 2 diabetes depending on whether the patient went onto insulin within the first 6 months from diagnosis. The calculator is highly accurate and out‐performs standard diagnostic criteria; a cut‐off of >60% achieves 92% sensitivity and 95% specificity for diagnosing MODY. Clinicians can set their probability thresholds for testing according to the resources available to them and numbers of patients they can feasibly test. A higher threshold will reduce the test burden but will miss cases. The calculator is used by clinicians in three out of four referrals to the Exeter Laboratory, and results in a higher MODY diagnostic rate compared to referrals where it has not been used (32% vs. 24%, unpublished data). The calculator is freely available to use online and as a mobile phone app (https://www.diabetesgenes.org/exeter‐diabetes‐app/ or search ‘Diabetes Diagnostics’ in the app store). Further work is ongoing to determine whether the addition of biomarkers such as islet autoantibody and C‐peptide status improves performance.

These models are less sensitive for insulin treated patients (and especially those at or close to diagnosis) and are designed and validated using White European cohorts. South Asian and Middle Eastern populations have a significantly higher prevalence of young‐onset type 2 diabetes where BMI thresholds for increased risk of insulin resistance are lower compared to White Europeans. This reduces the specificity of the calculator, and lower thresholds for age of diagnosis and BMI are required in ethnic groups to improve selection.^67^ The prevalence of MODY is likely to be similar in these ethnic groups and European populations, but much higher numbers of patients require testing to identify the same number of cases. Increased genetic testing in non‐White individuals is facilitating the development of population specific versions.

Clinical services can also improve their rates of MODY diagnosis through increased training, education and awareness. Examples of successful education initiatives include the national genetic diabetes nurse project, which trains diabetes specialist nurses to increase awareness of monogenic diabetes among healthcare professionals across the United Kingdom.^68^ A free online training course on monogenic diabetes is available^69^ and an annual 2 day face to face and virtual training course is run by the University of Exeter.^70^

**Figure 1:**
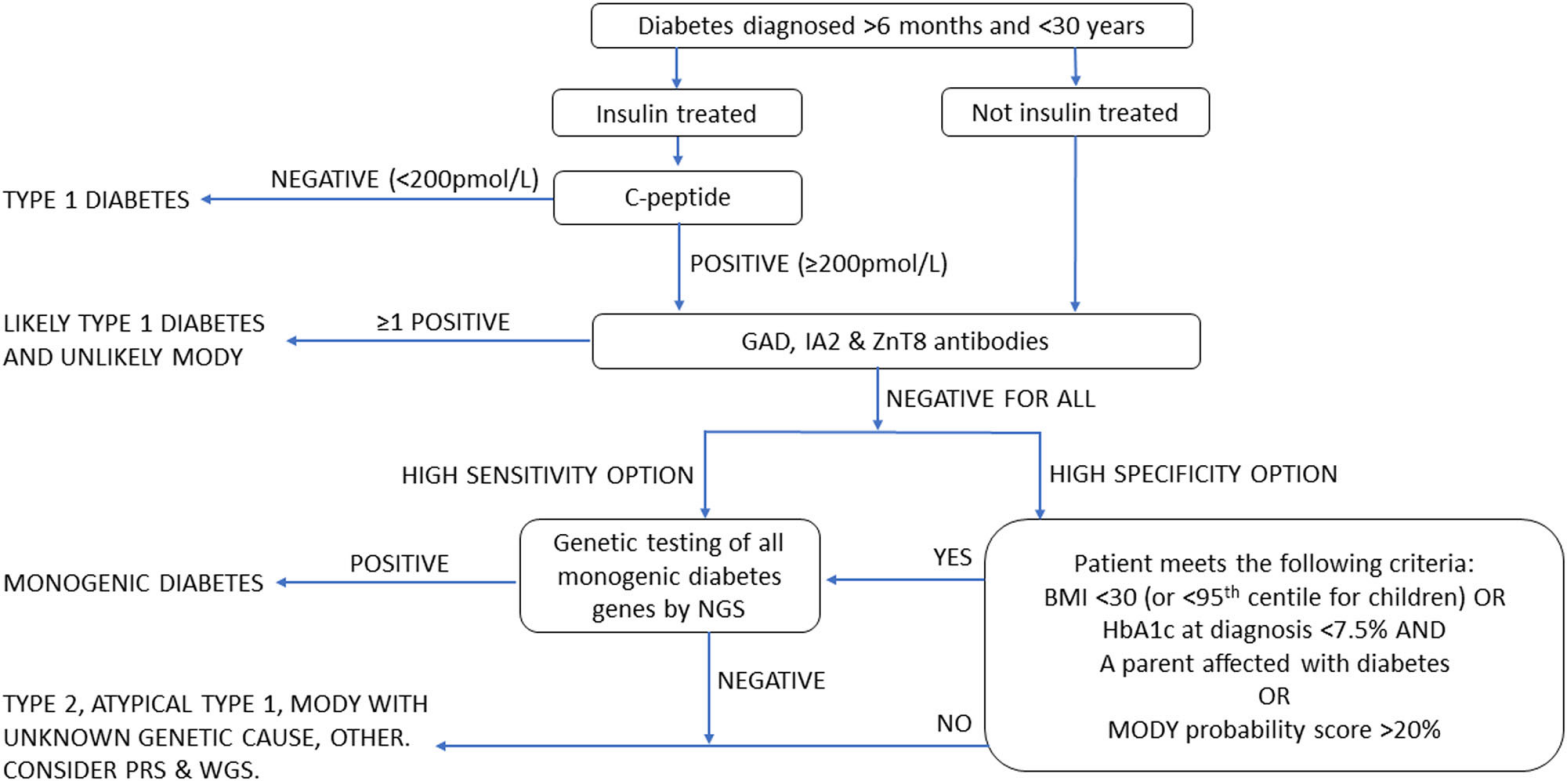
A strategy for identifying patients to refer for MODY testing. This strategy is based on the eligibility criteria for NHS funded MODY testing in England, available at https://www.diabetesgenes.org/tests‐for‐diabetes‐subtypes/guidelines‐for‐genetic‐testing‐in‐mody/ and within NHSE's Rare and inherited disease eligibility criteria document— https://www.england.nhs.uk/wp‐content/uploads/2018/08/Rare‐and‐inherited‐disease‐eligibility‐criteria‐2021‐22‐v2.pdf. These criteria were created using unpublished clinical information, family history and genetic testing data from the UK MODY registry at the Exeter Genomics Laboratory with the aim to achieve a MODY diagnosis in roughly one in every four patients referred for testing. BMI, body mass index; MODY, Maturity Onset Diabetes of the Young; NGS, next generation sequencing; PRS, polygenic risk score; WGS, whole genome sequencing. [Color figure can be viewed at wileyonlinelibrary.com]

MODY genetic testing is amenable to mainstreaming since a simple set of selection criteria can be used to identify patients for testing using a single assay that will test all known MODY genes. An example of a pathway for patient selection and testing is shown in Figure 1. This pathway suggests C‐peptide testing before antibodies since it is less expensive and noninvasive compared to antibody testing, but for practical reasons and patient convenience it may be preferable to take samples and test for C‐peptide and antibodies at the same time (particularly for newly diagnosed patients that are more likely to have detectable C‐peptide during the honeymoon period). The preference for sensitivity or specificity will depend upon the capacity to perform the tests—sacrificing sensitivity (i.e., more likely to miss some cases) for increased specificity (higher proportion of tests positive due to a higher positive predictive value) may be required if only limited numbers of tests can be performed. The high sensitivity option in Figure 1 will likely detect MODY in about one in six patients tested and identify ~99% of cases, whereas a high specificity pathway will make a diagnosis in about one in three patients but only identify ~50% of cases. Patients with a strong clinical suspicion of MODY but a negative genetic test may be recruited into whole genome sequencing (WGS) research studies to identify novel MODY genes or mutations in noncoding regions of known MODY genes. Measuring polygenic risk scores (PRS) for type 1 and type 2 diabetes will help to further refine the selection of patients most likely to have a monogenic cause for further study.^65^ Separate criteria based on FBG and HbA1c levels could be employed for selecting patients with a suspicion of GCK MODY for Sanger sequencing. Diabetes and paediatric teams might start by testing the very high probability cases to ensure a diagnosis which will lead to increased interest, motivation and enthusiasm for identifying more MODY cases once the positive impact on clinical management is recognized. It is important for clinicians to appreciate that identifying MODY is challenging due to the high prevalence of polygenic disease and it is acceptable for the majority their referrals to not receive a diagnosis (even in those with a high prior probability) and not to be disheartened by this.

Clinicians should be aware that patients with a diagnosis of MODY are still at population risk of developing polygenic autoimmune type 1 and insulin resistant type 2 diabetes. The incidence of childhood type 1 diabetes is increasing significantly in European populations,^71^ and more MODY patients are becoming obese and insulin resistant as a result of increasing global prevalence of obesity in young adults and children.^72^ Polygenic diabetes has been estimated to affect about 3%–4% of all patients with MODY (~3.5% with type 2 DM and 0.5% with type 1).^73^ This phenomenon is more easily observed in patients with GCK MODY since the insulin resistance or beta cell dysfunction/destruction causes blood glucose and HbA1c values to increase outside of the expected range. Having polygenic diabetes could result in a missed MODY diagnosis since the patient may not meet criteria for genetic testing. The mutation is likely to be detected only when a relative presents with a MODY phenotype and is referred for genetic testing. The specificity of BMI to discriminate type 2 diabetes from MODY will decrease with increasing obesity prevalence, and BMI cut‐offs within future paediatric MODY testing eligibility criteria will need to be relaxed or removed completely to prevent missed diagnoses.

## 4 WHAT IS THE PROCESS FOR GENETIC TESTING FOR MODY?

Clinicians should provide sufficient clinical information to the laboratory so that an appropriate testing strategy can be employed and to assist with the interpretation of detected genetic variants. The minimum information provided should be the patient's age of diagnosis, BMI (or weight centile if a child), HbA1c, treatment details (at diagnosis and current, and if insulin treated whether this was started within 6 months of diagnosis), family history (especially first‐degree relatives), antibody status, random nonfasting C‐peptide and ethnicity. Any additional extra‐pancreatic developmental conditions should be noted (e.g., structural renal disease, cerebellar signs, bilateral sensorineural hearing loss, skeletal or cardiac myopathy, lipodystrophy, optic atrophy) since they increase the likelihood of a syndromic subtype of monogenic diabetes.

The laboratory may ask for consent for the sample and clinical information to be stored in biobanks and used for future research studies and gene discovery work. The separation and storage of plasma from EDTA blood samples prior to DNA extraction provides material to study antibodies, C‐peptide and other biomarkers in MODY patients.

Mutation detection occurs through sequencing analysis for single nucleotide variants (SNVs) and small insertion/deletion/duplication variants (indels), and by copy number variant (CNV) analysis to detect partial and whole gene deletions that account for a small proportion of all mutations in MODY genes. Historically, laboratories would offer testing of a small number of genes by Sanger sequencing and MLPA, selecting the appropriate genes to test according to the clinical characteristics of the patient. This has been largely superseded by the advent of next generation sequencing (NGS) technology; it is now possible to test all of the known MODY genes (and other syndromic monogenic diabetes genes) in a single assay that will detect SNVs, indels and CNVs.^74^ This targeted NGS approach increases the diagnostic yield simply by testing more genes, but also identifies patients with rare syndromic forms of diabetes where the diagnosis was not suspected.^38^ This can be due to absence of the specific syndromic features (due to variable clinical expressivity, severity and penetrance) or unawareness by the clinician of the association between the features and a monogenic diabetes syndrome.

Targeted NGS simplifies the testing strategy since no prior assumptions about the specific genetic subtype are required; the clinician just needs to consider whether the patient has monogenic diabetes and refer for testing on that basis. One exception to this is the diagnosis of GCK MODY. These patients have a striking phenotype of persistent, stable, mild fasting hyperglycaemia from birth (FBG in range 5.5–8 mmol/L and HbA1c 5.5%–7.5% [40–60 mmol/mol]) that is asymptomatic and not altered through treatment. A high prevalence of GCK MODY is found in children with asymptomatic mild fasting hyperglycaemia and in nonobese women with gestational diabetes. Clinicians can identify large numbers of GCK MODY families simply by testing all GDM cases with a BMI of <25 and a fasting blood glucose of >5.5 mmol/L.^75^ The impact of a GCK MODY diagnosis on pregnancy management justifies the provision of a single gene testing option to enable rapid diagnosis and subsequent access to noninvasive foetal genotyping.

Genetic testing for MODY is available in many countries. The Genetic Testing Registry currently lists 65 laboratories across 18 different countries but this is not an exhaustive list.^76^ Genetic test costs for MODY will vary significantly between countries depending on the test methodologies employed and the funding source (e.g., government, insurance, private). As an example, genetic testing for patients in England is centrally funded by the National Health Service if eligibility criteria are met, with no charge to the referring clinician or patient. For all non‐NHS England requests there is a charge of 350 GBP for Sanger sequencing of the GCK gene (when a diagnosis of GCK MODY is suspected) and 650 GBP for targeted next generation sequencing of 52 monogenic (MODY and syndromic) diabetes genes. Clinicians should always seek testing using targeted NGS where possible to maximize the chance of diagnosing monogenic diabetes in their patient. Careful consideration of the NGS methodology and data analysis/interpretation processes used is required to avoid diagnostic errors.^77^ Laboratories performing NGS analysis must ensure that only genes known to cause MODY are analysed (although research/putative genes may be analysed but not included in the diagnostic report), and that variants are interpreted/classified correctly using appropriate guidelines to avoid a harmful misdiagnosis. MODY and syndromic diabetes genes should be tested by the NGS assay even if no syndrome is suspected since one in five referrals for MODY testing will have a mutation in a syndromic diabetes gene.^38^ CNV analysis must be performed to detect whole gene deletions of *HNF1B* that account for at least 50% of mutations in this gene, and to detect the small proportion of large deletions in other MODY genes. Clinicians should not accept a diagnosis of PAX4, BLK or KLF11 MODY since these are not causative genes^29^ and should query reports containing heterozygous pathogenic variants in genes causing recessively inherited disease (e.g., *WFS1*). For the *CEL*, *PDX1*, *INS*, *KCNJ11* and *ABCC8* genes, MODY is caused only by specific variants resulting in dominant‐negative/gain of function effects (see Table 1); null variants in these genes do not cause MODY. Expert‐led MODY gene and variant curation projects are ongoing via ClinGen to accurately classify MODY gene‐disease associations and the clinical significance of variants found within them.^31^

It is important for clinicians to remember that a genetic diagnosis has implications for the wider family, and not just the proband. Genetic testing should be offered to all relatives with diabetes to confirm a MODY diagnosis before considering changes in their clinical management. It is inappropriate to assume a MODY diagnosis in family members since phenocopies are common due to the high population prevalence of type 1 and type 2 diabetes. Healthy family members at risk of inheriting the variant can opt either for presymptomatic genetic testing or annual biochemical screening for diabetes and having a genetic test once a diagnosis of diabetes has been made.^60^ Genetic counselling should be offered (except for GCK MODY) because the diabetes is not congenital and is not 100% penetrant and is essential when a mutation in a syndromic diabetes gene has been unexpectedly identified.

Clinicians should receive a fully interpretative report from the laboratory that states clearly whether a genetic diagnosis of MODY has been made, details of any actionable variants identified and any evidence to support variant classification, possible implications for clinical management, implications/risks for family members, recommended additional clinical investigations or family member testing, and details of the test methodology used. The report format must be clear and free of complex jargon so that it is understandable to both patients and clinicians.

## 5 CONCLUSIONS

Our knowledge of MODY has improved significantly since the first description of the disorder in 1974. And whilst efforts continue to discover new genes, increase our understanding of the disease mechanisms and determine effective treatments, most patients with MODY remain misdiagnosed. Through education and training initiatives we can increase awareness of the clinical and health economic benefits to diagnosing MODY, motivating clinicians to increase diagnostic rates in their clinics. And through improved selection criteria using probability models and biomarkers we can implement systematic approaches to help clinicians identify patients for testing, ultimately aiming to mainstream MODY diagnosis within primary and secondary healthcare settings. This will help to increase access to MODY genetic testing and better clinical care, and reduce the numbers of patients with a missed diagnosis. Further work is needed to refine prediction tools, especially for MODY diagnosis in newly diagnosed patients on insulin or patients with young type 2 patients, and also individuals from non‐White ethnic groups. The incorporation of biomarkers and polygenic risk scores into existing models will no doubt improve their performance. Advances in DNA sequencing technology have changed the paradigm of genetic testing by simplifying testing strategies and increasing diagnostic yield, but this shifts some of the diagnostic burden from clinician to laboratory scientist. As the test complexity increases, correct genetic testing, variant interpretation and reporting is essential to avoid patient harm.

## CONFLICT OF INTEREST

The authors declare no conflict of interest.

## Data Availability

Data sharing is not applicable to this article as no new data were created or analysed in this study.
